# The Effect of Health Insurance on the Utilization of Health Services: A Systematic Review and Meta-Analysis

**DOI:** 10.31661/gmj.v8i0.1411

**Published:** 2019-10-14

**Authors:** Elham Shami, Jafar Sadegh Tabrizi, Shirin Nosratnejad

**Affiliations:** ^1^Iranian Center of Excellence for Health Management, School of Management and Medical Informatics, Tabriz University of Medical Sciences, Tabriz, Iran; ^2^Department of Health Services Management, Tabriz Health Services Management Research Center, Tabriz University of Medical Sciences, Tabriz, Iran; ^3^Iranian Center of Excellence in Health Services Management, Department of Health Management and Economics, School of Management and Medical Informatics, Tabriz University of Medical Sciences, Tabriz, Iran

**Keywords:** Health Care Utilization, Health Insurance, Inpatient, Outpatient Care

## Abstract

Insurance organizations are among the most influential organizations in the health system, which can lead to healthcare efficiency and patient satisfaction in case they are increasingly accessed. The main purpose of the present systematic review was to examine the effect of health insurance on the utilization of health services and also to examine the factors affecting it. The present study was a systematic review that aimed to examine the effect of health insurance on the utilization of health care services. The study was conducted in 2016 using Scopus, PubMed, Web of Science, Science Direct, and ProQuest databases. We examined the utilization rate of health insurance in insured people. The inclusion and exclusion criteria were included based on review and meta-analysis purposes. The utilization of health services increased for inpatient and outpatient services. The utilization rate of inpatient services increased by 0.51% whereas the utilization rate of outpatient services increased by 1.26%. We classified the variables affecting the utilization rate of insurance into three main categories and sub-categories: demographic variables of the household, socioeconomic status, and health status. Our study showed that insured people increased the utilization rate of health services, depending on the type of health services. Thus, health policymakers should consider the community’s health insurance as a priority for health programs. For now, implementing universal health insurance is a good solution.

## Introduction

One of the most important strategies for achieving social equality in health systems in countries is the easy access of the population to the required health services[1, 2]. One of the main goals of policymakers in the health and treatment sector of each country is to facilitate access and utilize health services [3-5]. An effective factor for access to health services is universal health insurance. The main mission of health insurance is to remove major obstacles toward access to health care, especially for vulnerable groups in the community. With the increase in health costs, more people need extensive assistance for insurance. Health insurance protects those with limited financial resources or those who need more health services. According to the 1999 World Health Report, there is a need for a prepayment mechanism to gain effective access to costly individual care. The prepayment mechanism also allows financial distress distribution among members of a fund. Risk pooling from healthy to sick, wealthy to poor, and young to elderly will lead to utilizing all health services if needed. Thus, uninsured people will cause problems when they are sick [1]. The importance of health insurance is evaluated by estimating the negative outcomes of no insurance. There is a probability that those without insurance will ignore their health needs more than those who are insured. Those without health insurance are more likely to face various obstacles when receiving health services. Studies have shown that parents’ insurance status affects children’s utilization of health services. Health insurance will also change the utilization rate with increasing access to health services [2]. Studies have shown that having health insurance increases the utilization rate of health services [6, 7], i.e., increases the likelihood of using health services in the event of diseases, as having insurance increases the utilization rate of inpatient services up to 40% [8]. Moreover, even in the children’s age group, no insurance leads to a lack of access to required health services [9-13]. There is a gap to examine the extent to which health insurance will increase the utilization of health services. Therefore, the present study investigated the effect of health insurance on the utilization of outpatient and inpatient services, as well as the variables affecting the utilization of such services.

## Search Strategies

### Information Resources

Our study was conducted in 2016 by using Scopus, PubMed, Web of Science, Science Direct, and ProQuest databases. The research question was, “Does health insurance affect health service utilization?”

### Search Strategy and Databases

A general search was conducted on the subject of “The Effect of Insurance on Utilization of Health Services.” The keywords in this systematic review were obtained using Mesh in the PubMed database through advanced searches and were examined by qualified individuals. The keywords include health care utilization, healthcare utilization, and health insurance (standard error [SD]-1). Afterward, the main and systematic searches were conducted using the keywords obtained from the given databases (Scopus, Web of Science, PubMed, Science Direct, and ProQuest) and/or with “AND” and “OR” operators. The search period was from October 23 to December 15, 2015. The search strategy is presented in Table-1. The studies were obtained free of charge from the databases or the central library of the Tabriz University of Medical Sciences. If there was no paper file, some of the papers were also obtained via e-mail.

### Eligibility Criteria

Primary studies that assessed the effects of health insurance on the utilization of health care were eligible for inclusion. Studies were considered eligible for inclusion if they:

1. Investigated the effect of utilization in the usual household

2. Determined the effect of health insurance on health service utilization

3. Compared to the utilization rate before and after obtaining insurance

4. Were conducted only in English and with no time limit

5. Evaluated the impact of health insurance on diseases and specific cases

### Quality Appraisal of the Studies

According to the type of the selected papers, an appropriate tool was used for their evaluation. In this study, given that all the papers were quantitative and mostly surveyed, descriptive or cross-sectional, the quality was evaluated by the STROBE checklist. The STROBE checklist consists of 22 sections including the definition of title and abstract, introduction, method, sample, variable, data sources, measurement, bias, sample size, quantitative variables, sensitivity analysis method, results, discussion, study limitations, and study results, with each section having separate questions.

### Data Extraction

At this stage, the data in the studies was extracted, which included the country of the study, year of conducting the study, name of the authors of the study, sample size, statistical population, type of the sample (household or individual), type of the study, data collection method, and effects of insurance on the utilization of services.

### Data Analysis and Synthesis

We used two approaches of meta-analysis and counting vote to analyze the data extracted from the studies.

- Meta-analysis: to analyze the effect of health insurance on health service utilization

- Counting votes: to analyze the factors affecting health service utilization

### Meta-Analysis

Meta-analysis involves combining the data and results obtained from studies with a systematic review using statistical methods; thus, meta-analysis consists of two parts: estimating the effect size and estimating the weight of the effect size. In this study, the meta-analysis was performed on the results using STATA 12 software (Stata Corporation, College Station, TX, USA), and the required graphs were plotted.

### Estimating Effect Size

In the meta-analysis, the effect size was the health service utilization through insurance which was reported in the obtained studies in various ways such as the percentage of utilization, mean utilization before and after having insurance and/or among those insured compared to uninsured ones. In this study, the effect size was estimated in the form of the rate of changes in the utilization rate after having insurance using the following formula: The rate of utilization before having insurance / (the utilization rate before having insurance – the utilization rate after having insurance) = the effect size of each study

### Estimating Standard Error of the Effect Size

The weight of the effect size is the standard error (SE) related to the effect size of each study, which, in various SE papers, SD and/or confidence interval (CI) has been reported. All the numbers were converted into SE according to the corresponding formulas.

### Vote Counting

A descriptive approach of vote counting was used to determine the factors affecting the utilization rate of services. In the present study, the variables affecting the rate of health service utilization, as expressed in various papers, were identified. The obtained variables were classified into three groups of variables including those that had a significant positive effect on the utilization rate of services, those that had a significant negative impact on the utilization rate of services, and those that had no significant effect on the utilization rate of services. In the next stage, the number of variables was counted in the studies.

## Results

### Search Results

**Figure 1:**
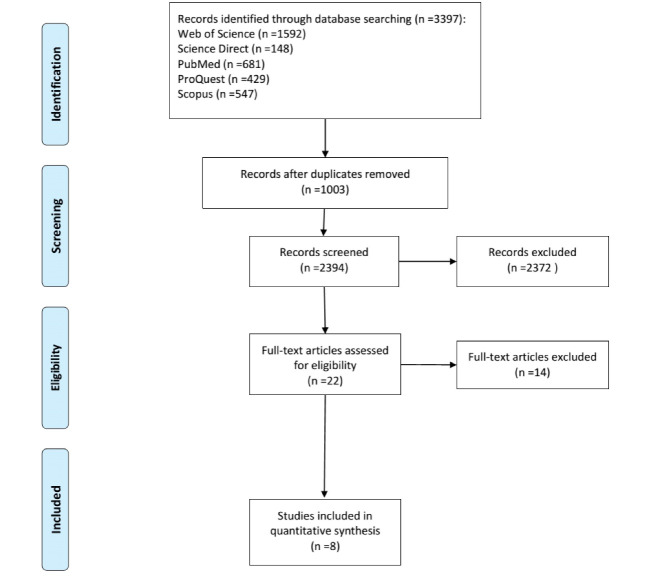
The PRISMA flow diagram of reviewing the articles for health care utilization

In the preliminary search, 3626 papers were obtained and entered into the software EndNote. The studies were screened according to their titles and abstracts, respectively. After reviewing the full texts, eight papers were finally selected according to the inclusion and exclusion criteria (Figure-1).

### Quality Appraisal of the Studies

The selected studies were evaluated qualitatively using the STROBE checklist. Studies by Neena *et al.* (2012), Stephen *et al.* (2009), and Beth Hahn (1994) had the highest quality in this evaluation (SD-2) [14-16].

### Characteristics of the Included Studies

The obtained studies were conducted in countries such as the United States, Canada, China, Jamaica, South Korea, and India. Studies were descriptive surveys, in which the sample size varied from 297 to 12270 households (Table-2).

### Meta-Analysis Results

**Figure 2:**
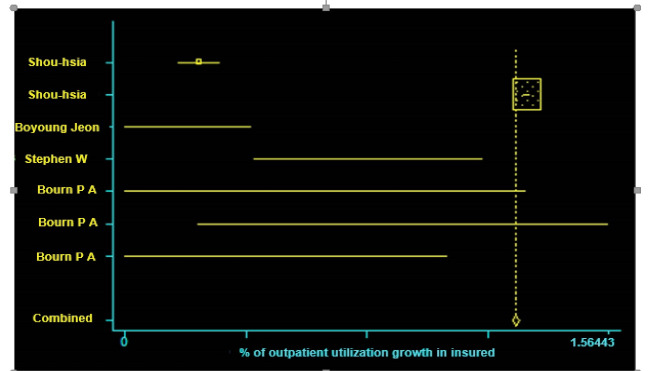
The result of meta-analysis for health insurance and its effect on outpatient service utilization.

**Figure 3:**
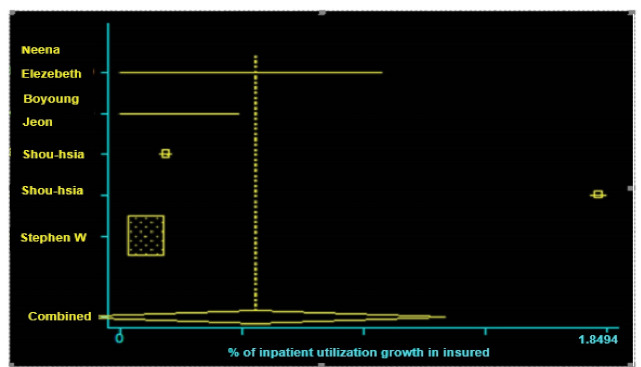
The result of meta-analysis for health insurance and its effect on inpatient service utilization

In the present study, among the eight selected studies, four studies had meta-analysis inclusion criteria. These studies included information on the rate of utilization before and after having insurance, standard error in the studied subjects, or the minimum rate of utilization comparatively [10, 16, 17, 18]. However, four of the studies did not have the meta-analysis inclusion criteria because the standard error rate was not specified and could not be calculated [14, 15, 19, 20]. According to the meta-analysis results, the utilization rate of inpatient services with a standard error of 95% and a confidence interval of -0.206-0.235 increased by 0.51%, whereas the utilization rate of outpatient services with a standard error of 95% and a confidence interval of 1.254-1.277 increased by 1.26% ( Table-3, Figures-2 and 3).

### Determination of the Effective Factors

All the variables affecting the health service utilization were examined using the vote counting method. We classified the variables into three main categories and several sub-categories: 1. Household demographic variables (gender, age, household size, and maternal age), 2. Economic-social status (residence, education, household income, mother's occupation, household head status, socioeconomic conditions, etc.), and 3. Health status (birth weight, health status, medical expenses, health needs, and chronic disease). The results showed that age was one of the variables affecting the utilization rate of services in the group of demographic variables so that a significant and positive effect was proven in four out of the total five studies. Education and social class were the variables affecting the utilization in the socio-economic status so that a significant and positive effect was proven in two out of the three studies (Table-4).

## Discussion

The present study was a systematic review aiming to examine the effect of health insurance on the utilization of health services and also to investigate the factors influencing the utilization of such services. It can be said that the study is among the early systematic reviews in this field. The results of the meta-analysis of the studies showed an increase in the insured people’s utilization of inpatient and outpatient health services. Accordingly, the utilization rate of inpatient services increased by 0.51% whereas the utilization rate of outpatient services increased by 1.26%. The studies showed that with insurance, the population would receive more voluntary, emergency and inpatient services, which would be much higher in voluntary services than in emergency and inpatient services [16, 21, 22]. This increase is because the socioeconomic variables and health conditions of both insured and uninsured people were controlled [8, 13, 16]. The highest rate of the insured people’s utilization of inpatient and outpatient health services in the study by Stephen *et al.* (2013) was related to the utilization of emergency services. In their study, the reason for the increased utilization rate of emergency services is low socioeconomic status and lack of payment for health service utilization [22-24]. Accordingly, the lack of health insurance is the first obstacle in receiving primary care in the United States, which has worsened the condition of patients and increased the demand for emergency services [25, 26]. This is while the lowest rate of utilization in the study by Jeon *et al.* (2013) was shown with an increase of 0.07 in the rate of inpatient services [27, 28]. The study was about those with private insurance; private insurance exclusively considers outpatient services and inpatient services are not considered [10]. The existing studies have examined utilization of health services in three types of health insurance (universal health insurance, social insurance, and private insurance), which show that utilization of services is higher for those with universal insurance than for those with social insurance. The studies also indicate that utilization of services for social health insurance is more than that of private health insurance. The reason for such differences could be the inherent difference between these three types of insurances [10, 12, 29]. In universal health insurance, the government insures all individuals, without considering the financial capability of individuals, for an appropriate service package, and the health costs of the poor are paid by government revenues so that all individuals, regardless of financial and socioeconomic status, have health insurance [30, 31]. Social health insurance covers individuals based on their occupational category or compulsorily region of residence; thus, those who do not meet the terms are not covered by health insurance, and uninsured individuals may have less health service utilization. As opposed to the two previous types of insurance, private insurance leads to less health service utilization; the reason would be the low coverage of this type of health insurance as it only covers outpatient services [32, 33]. Private health insurance is mostly purchased by those who are wealthy and healthy and those who are covered by universal health insurance and are willing to utilize private health insurance as supplementary health insurance because they can pay for it [31]. Private health insurance also does not cover high-risk individuals [34, 35]. The most important variables affecting the utilization of health services in addition to health insurance are age, education, socioeconomic conditions and health status [36]. The elderly were more referred to health centers for outpatient and inpatient services [37]. Admission days were also higher for those who were older. Most of the cases were in the age group of 60-74-years-old. Moreover, those with lower income and education had less access to health services [38]. The results showed that lower socioeconomic status led to more referral at more advanced stages of the disease. The study results also showed that health insurance increased the amount of care received and access to health care [35-38].

## Study Limitations

Few studies are focused on the direct effect of insurance on health service utilization. Moreover, due to the existence of different health insurances and mechanisms in various countries, the analysis of the results became complicated and thus meta-analysis was not performed on some of the studies.

## Conclusion

our study showed that health insurance increased access to the utilization of health services. Accordingly, the utilization of outpatient and inpatient care services increased by 1.26 and 0.51, respectively. Moreover, given that universal health insurance provides patients with the greatest access to health services and leads to the highest utilization rate, all the people were covered by universal health insurance regardless of their socioeconomic status. Therefore, governments should take measures to move towards universal health insurance.

## Acknowledgment

This study was supported by the Research Chancellor of the Tabriz University of Medical Sciences (granny number: 5/6807). The authors are grateful to all the staff at the health department of the Tabriz University of Medical Sciences.

## Conflict of Interest

The authors declare that they have no conflicts of interest.

**Table 1:** Inclusion and Exclusion Criteria

**Criterion**	**Inclusion**	**Exclusion**
**Population**	General population	Patient and specific populations
**Intervention**	Health insurance effecting	Other studies
**Study design**	The comparative studies that have examined the extent of utilization before and after the insurance or among insured with uninsured ones	The effect of health insurance on utilization for diseases and services and specific populations
**Language**	English	Other languages

**Table 2:** Characteristics of Included Articles

**Study**	**Country**	**Sample size**	**Type of study**	**Data collection method**	**Population**	**Outpatient utilization growth rates**	**Inpatient utilization growth rates**
Shou *et al*. (1997)	China	1021	Survey	Questionnaire	Individual	1.82	1.296
Thomas *et al*. (2003)	United State	14000	Review	Secondary data	Individual Household	-	2.43
Tracy *et al*.(2003)	United State	754	Survey	Questionnaire	Individual	-	0.18
Stephen *et al*. (2009)	Canada	1165	Survey	Questionnaire	Individual Household	4.66	7.782
Bourne *et al*.(2010)	Jamaica	6783	Survey	Questionnaire	Households	-	0.6
Cassedy *et al*. (2008)	United State	12 270	Survey	Questionnaire	Households	-	1.8
Jeon *et al*. (2011)	South Korea	9512	Survey	Questionnaire	Individual Household	0.07	0.1
Neena *et al*. (2012)	India	297	Survey	Questionnaire	Household	0.35	-

**Table 3:** The Results of Meta-Analysis of the Included Studies (Percent of Utilization Growth Rates)

**Variable**	**Method**	**Pooled estimate**	**95% confidence interval**	**P-value**	**Ref**
The rate of utilization from inpatient at insured persons	Random	0.515	-0.206-1.235	0.000	[10, 16-18]
The rate of utilization from outpatient at insured persons	Fixed	1.265	1.254-1.277	0.000	[14, 15, 19, 20]

**Table 4:** Effective Factors on Health Care Utilization

**Variables**		**Neena** ***et al*** **. (2012)**	**Erin** ***et al***. **(2006)**	**Stephen** ***et al***. **(2009)**	**Bourne** ***et al***. **(2010)**	**Beth Hahn** **(1994)**	**Tracy** ***et al*** **. (2003)**	**Jeon** ***et al*** **. (2011)**	**Shou** ***et al*** **. (1997)**
**Demographic**	Sex	⬆	-	-	-	-	-	-	-
	Age	⬆	⬆⬆	-	⬆	⬆⬆	-	-	-
	Household size	⬆	-	-	-	-	-	-	-
	Maternal age	-	⬆	-	-	-	-	-	-
**Socioeconomic statue**	Area of resident	-	-	-	⬆⬆	-	-	-	-
	Education	-	⬆	-	⬆⬆	⬆⬆	-	-	-
	Household income	-	⬆	-	-	⬆⬆	-	-	-
	Mother employment	-	⬆	-	-	-	-	-	-
	Socioeconomic status (Social class)	⬆⬆	⬆⬆	-	⬆⬆	-	-	-	-
	Employed household head	⬆	-	-	-	-	-	⬆⬆	-
**Health status**	Low birth weight	-	⬆	-	-	-	-	-	-
	Health condition (self-related health statues)	-	-	-	⬆⬆	⬆⬆	-	-	-
	Medical expenditure (Outpatient expenditure)	-	-	-	⬆⬆	⬆⬆	-	-	-
	Health care needs	-	-	-	⬆⬆	-	-	-	-
	Having chronic disease	⬆⬆	-	-	-	-	-	-	-

↑↑ The effect of the variable is positive and significant; ↓↓ The effect of the variable is negative and significant; ↓ The effect of the variable is negative and non-significant; ↑The effect of the variable is positive and non-significant
